# Risk factors associated with surgical site infections after thoracic or lumbar surgery: a 6-year single centre prospective cohort study

**DOI:** 10.1186/s13018-021-02418-1

**Published:** 2021-04-15

**Authors:** Vera Spatenkova, Ondrej Bradac, Zdenek Jindrisek, Jan Hradil, Daniela Fackova, Milada Halacova

**Affiliations:** 1 Neurocenter, Neurointensive Care Unit, Regional Hospital, Husova 357/10, 46063 Liberec, Czech Republic; 2 Department of Neurosurgery, Military University Hospital and First Medical School, Charles University, Prague, Czech Republic; 3 Neurocenter, Department of Neurosurgery, Regional Hospital, Liberec, Czech Republic; 4 Department of Clinical microbiology and immunology, Antibiotic Centre, Regional Hospital, Liberec, Czech Republic; 5 Department of Clinical Pharmacology, Na Homolce Hospital, Prague, Czech Republic

**Keywords:** Surgical site infection, Preventive infection protocol, Wound complications, Antibiotic prophylaxis, Spine surgery

## Abstract

**Background:**

Surgical site infection (SSI) is a risk in every operation. Infections negatively impact patient morbidity and mortality and increase financial demands. The aim of this study was to analyse SSI and its risk factors in patients after thoracic or lumbar spine surgery.

**Methods:**

A six-year single-centre prospective observational cohort study monitored the incidence of SSI in 274 patients who received planned thoracic or lumbar spinal surgery for degenerative disease, trauma, or tumour. They were monitored for up to 30 days postoperatively and again after 1 year. All patients received short antibiotic prophylaxis and stayed in the eight-bed neurointensive care unit (NICU) during the immediate postoperative period. Risk factors for SSI were sought using multivariate logistic regression analysis.

**Results:**

We recorded 22 incidences of SSI (8.03%; superficial 5.84%, deep 1.82%, and organ 0.36%). Comparing patients with and without SSI, there were no differences in age (*p*=0.374), gender (*p*=0.545), body mass index (*p*=0.878), spine diagnosis (*p*=0.745), number of vertebrae (*p*=0.786), spine localization (*p*=0.808), implant use (*p*=0.428), American Society of Anesthesiologists (ASA) Score (*p*=0.752), urine catheterization (*p*=0.423), drainage (*p*=0.498), corticosteroid use (*p*=0.409), transfusion (*p*=0.262), ulcer prophylaxis (*p*=0.409) and diabetes mellitus (*p*=0.811). The SSI group had longer NICU stays (*p*=0.043) and more non-infectious hospital wound complications (*p*<0.001). SSI risk factors according to our multivariate logistic regression analysis were hospital wound complications (OR 20.40, 95% CI 7.32–56.85, *p*<0.001) and warm season (OR 2.92, 95% CI 1.03–8.27, *p*=0.044).

**Conclusions:**

Contrary to the prevailing literature, our study did not identify corticosteroids, diabetes mellitus, or transfusions as risk factors for the development of SSI. Only wound complications and warm seasons were significantly associated with SSI development according to our multivariate regression analysis.

## Background

Every surgery carries a risk of SSI, a complication which negatively impacts patient morbidity and mortality, increases financial demands by prolonging hospital stay, and may require further antibiotics and surgical procedures. SSIs are a significant group of healthcare-associated infections with high preventability [1, 2]. Prudent preventive strategies have an important role in increasing postoperative patient safety and can limit the incidence of multidrug resistant strains. The elimination of this complication is a priority in all surgical management and is particularly important in spine operations, where these risks are heightened due to the frequent use of metallic implants, the nearby localization of the spinal cord, and the load-bearing function of the spine. The incidence of SSI in spine surgery varies from 2 to 13% according to literature of varying quality and methodology [3–10]. A protective protocol includes many strategies for reducing the risk of developing an SSI. This involves maintaining correct antibiotic prophylaxis, and proper hygiene throughout all stages of surgery and general care, not only in the operating theatre but crucially during the postoperative period until the wound has healed [1, 2].

The aim of this study was to identify and analyse SSI in accordance with international definitions, and to search for risk factors associated with its onset in patients who had undergone thoracic or lumbar spine surgery.

## Method

A 6-year single-centre prospective observational cohort study was conducted in the Neurocenter at the 900-bed Liberec Regional Hospital from 1 January 2005 to 31 December 2010. The incidence of SSI was monitored in 274 who fulfilled our inclusion criteria. These criteria were (1) planned operation; (2) thoracic or lumbar localization; (3) degenerative disease, trauma, or tumour; (4) short antibiotic prophylaxis defined as antibiotic administration before surgery and during long operations; and (5) patients who were recommended by neurosurgeons or anaesthetists for a postoperative stay in the eight-bed Neuro-intensive Care Unit (NICU) which has a multi-modal preventive infection control and normoglycemia protocol. Exclusion criteria were as follows: (1) primary infection of the spine and (2) prolonged antibiotic prophylaxis defined as continued antibiotic administration before and/or after the operation.

SSIs were classified according to Horan et al. [11] as (1) superficial: skin and subcutaneous tissue; (2) deep: fascia and muscle; and (3) organ: organs and spaces. For superficial SSI without microbiology we included borderline cases. These were defined as either a large seroma, or spontaneous dehiscence with secretion and high CRP. Borderline cases were treated with local or systemic antibiotic therapy. The incidence of SSI was monitored for up to 30 days postoperatively and again after 1 year. The following prevention protocols were adhered to in order to conduct SSI analysis. Hygiene rules: (1) hand hygiene before and after patient contact and each procedure; (2) masks, surgical caps, sterile surgical gowns, and sterile insertion of systems in invasive medical procedures; (3) disinfection soap before entering the operating theatre; (4) principles for drainage and tubes: single-use products, closed systems, minimal necessary duration, minimal and only necessary disconnection using the port system, and regular and irregular exchange according to protocol; and (5) surgical wound fully covered and dry.

Antibiotic prophylaxis: We mainly used two types of antibiotics without rotation. The first choice antibiotic was cefazolin; in case of allergy, clindamycin was used.

(1) Short prophylaxis: Administered before and during the operation, without prolonging use after the operation. (2) Doses: Intravenous administration of the appropriate dose of antibiotics. In patients up to 120 kg either 2 g of cefazolin or 600 mg of clindamycin, over 120 kg either 3 g of cefazolin or 900–1200 mg of clindamycin, with repeated administration during high blood loss (over 1.5 l blood). (3) Timing: The correct timing before incision (30–60 min) and perioperative administration at the correct interval (cefazolin at 4 h, clindamycin without need for further administration) [1]. We studied the following risk factors of SSI: (1) parameters associated with operations (localization, number of vertebrae, reoperation, time of operation, use of graft and implant, ASA Score); (2) use of medical devices: drainage, airways, mechanical ventilation, and catheters (artery, central venous, urine); (3) administration of corticosteroids (methylprednisolone, hydrocortisone); (4) transfusions, blood loss, and haemoglobin; (5) ulcer prophylaxis; (6) diabetes mellitus; (7) Acute Physiology and Chronic Health Evaluation (APACHE) II score on admission; (8) C-reactive protein (CRP); (9) length of stay in the NICU and in our hospital; (10) non-infectious hospital wound complications; and (11) warm season (June, July, August).

### Statistical analysis

Parametric *t*-tests or non-parametric Mann-Whitney *U* tests were used for comparison of continuous parameters. Comparison of categorical parameters was carried out using chi-square or Fisher’s tests as appropriate. Univariate logistic regression was used for identifying prognostic factors of wound complications. Factors from our univariate analysis that met the significance threshold of *p* <0.1 were used for multivariate regression analysis; factors with *p* value <0.1 were left in the model. *P* values of less than 0.05 were considered significant. STATISTICA 13.2 (TIBCO Software Inc., Palo Alto, CA, USA) software was used for statistical analyses.

The study was conducted after the approval of the Regional Hospital Ethics Committee for Multicentric Clinical Trials.

## Results

Of 286 consecutive patients treated at our centre, 274 met our inclusion criteria and were included in our study. We excluded twelve patients due to their prolonged antibiotic prophylaxis following the operation. The results of short antibiotic prophylaxis are shown in Table 1.

**Table 1:** Antibiotic prophylaxis

Parameter	Unit	Total population ***N***=274	Control group ***N***=252	SSI ***N***=22	***p*** value
1-Dose operation	pts	145 (52.92%)	133 (52.78%)	12 (54.55%)	
2-Dose operation	pts	122 (44.53%)	112 (44.44%)	10 (45.45%)	0.731
3-Dose operation	pts	7 (2.55%)	7 (2.78%)	0 (0.00%)	
Cefazolin	pts	245 (89.42%)	225 (89.29%)	20 (90.91%)	0.812
Clindamycin	pts	27 (9.85%)	25 (9.92%)	2 (9.09%)	0.900
Amoxicillin-clavulanate	pts	2 (0.73%)	2 (0.79%)	0 (0.00%)	0.675

Over 6 years, we recorded 22 incidences of SSI (8.03%), the majority were superficial (5.84%), and a few were deep (1.82%) or organ (0.36%). When patients with SSI were compared with the control group, there were no significant differences in demographic data, diabetes mellitus, or ulcer prophylaxis (Table 2). There was also no difference concerning corticosteroid use. The mean duration of corticosteroid use was 2.14±1.17 days. Results associated with operations are shown in Table 3. No differences were found in localization, number of vertebrae, duration of operation, or any other parameter associated with operations. Similarly, non-significant results were found in parameters associated with the immediate postoperative period in the NICU.

**Table 2:** Demographic and clinical data of spine surgery patients

Parameter	Unit	Total population	Control group	SSI	***p*** value
Number total	pts	274 (100%)	252 (91.97%)	22 (8.03%)	
2005	pts	37 (13.50%)	33 (13.10%)	4 (18.18%)	
2006	pts	46 (16.79%)	41 (16.27%)	5 (22.73%)	
2007	pts	34 (12.41%)	30 (11.90%)	4 (18.18%)	0.746
2008	pts	47 (47.15%)	44 (17.46%)	3 (13.64%)	
2009	pts	57 (20.80%)	54 (21.43%)	3 (13.64%)	
2010	pts	53 (19.34%)	50 (19.84%)	3 (13.64%)	
January	pts	31 (11.31%)	29 (11.51%)	2 (9.09%)	
February	pts	18 (6.57%)	15 (5.95%)	3 (13.64%)	
March	pts	36 (13.14%)	35 (13.89%)	1 (4.55%)	
April	pts	25 (9.12%)	23 (9.13%)	2 (9.09%)	
May	pts	20 (7.30%)	19 (7.54%)	1 (4.55%)	
June	pts	22 (8.03%)	20 (7.94%)	2 (9.09%)	
July	pts	15 (5.47%)	12 (4.76%)	3 (13.64%)	
August	pts	27 (9.85%)	23 (9.13%)	4 (18.18%)	
September	pts	22 (8.03%)	21 (8.33%)	1 (4.55%)	
October	pts	27 (9.85%)	25 (9.92%)	2 (9.09%)	
November	pts	21 (7.66%)	20 (7.94%)	1 (4.55%)	
December	pts	10 (3.65%)	10 (3.97%)	0 (0.00%)	
Cold season	pts	210 (76.64%)	197 (78.17%)	13 (59.09%)	
Warm season	pts	64 (23.36%)	55 (21.83%)	9 (40.91%)	**0.042**
Age	pts		54.06±12.89	56.59±13.18	0.374
Male	pts	145 (52.92%)	132 (52.38%)	13 (59.09%)	0.545
Weight	kg		80.72±14.94	79.36±15.05	0.668
BMI			27.48±4.00	27.69±4.17	0.878
NICU stay	day		1.61±1.10	1.32±1.51	**0.043**
Hospital stay	day		8.65±5.92	11.55±6.35	0.094
Spine diagnoses					
Degenerative	pts	247 (90.15%)	227 (90.08%)	20 (90.91%)	
Tumour	pts	21 (7.66%)	19 (7.54%)	2 (9.09%)	0.745
Trauma	pts	6 (2.19%)	6 (2.19%)	0 (0.00%)	
Diabetes mellitus	pts	33 (12.04%)	30 (11.90%)	3 (13.64%)	0.811
Ulcer prophylaxis	pts	56 (20.44%)	53 (21.03%)	3 (13.64%)	0.409
Omeprazole	pts	50 (18.25%)	47 (18.65%)	3 (13.64%)	0.559

*BMI* body mass index, *NICU* neurointensive care unit. Warm season—June to August; cold season—January to May, September to December

**Table 3:** Characteristics of spine surgery

Operation	Unit	Total population ***N***=274	Control group ***N***=252	SSI ***N***=22	***p*** value
Localization					
Th	pts	26 (9.49%)	23 (9.13%)	3 (13.64%)	
Th-L	pts	2 (0.73%)	2 (0.79%)	0 (0.00%)	0.808
L	pts	130 (47.45%)	118 (46.83%)	12 (54.55%)	
LS	pts	116 (42.34%)	109 (43.25%)	7 (31.82%)	
Number of vertebrae			2.30±0.90	2.36±1.10	0.786
0–2	pts	202 (73.72%)	16 (66.67%)	199 (71.07%)	
3–4	pts	62 (22.63%)	57 (22.62%)	5 (22.73%)	0.972
5 and more	pts	10 (3.65%)	9 (3.57%)	1 (4.55%)	
ASA score			2.14±0.68	2.18±0.71	0.752
Reoperation	pts	27 (9.85%)	27 (10.71%)	0 (0.00%)	0.106
Time of operation	minutes		184.16±72.23	173.86±81.48	0.547
Operation access					
Anterior	pts	7 (2.56%)	1 (4.17%)	7 (2.51%)	
Posterior	pts	261 (95.60%)	240 (87.91%)	6 (3.395%)	0.938
Graft	pts	5 (1.82%)	5 (1.98%)	0 (0.00%)	0.505
Implant	pts	239 (87.23%)	221 (87.70%)	18 (81.82%)	0.428
Drainage					
Redon	pts	258 (94.16%)	238 (94.44%)	20 (90.91%)	0.498
One drainage	pts	61 (23.64%)	57 (23.95%)	4 (20.00%)	0.690
Two and more drainage	pts	197 (76.36%)	181 (76.05%)	16 (80.00%)	
Transfusions	pts	49 (17.88%)	47 (18.65%)	2 (9.09%)	0.262
Blood loss	ml		1027.82±968.97	747.37±1234.82	0.106
Haemoglobin			103.45±20.05	105.36±20.50	0.548
Corticoids	pts	26 (9.49%)	25 (9.92%)	1 (4.55%)	0.409
Methylprednisolone	pts	5 (1.82%)	5 (1.82%)	0 (0.00%)	0.505
Hydrocortisone	pts	18 (6.57%)	17 (6.75%)	1 (4.55%)	0.689
Postoperative NICU					
TISS on admission			57.21±1.01	57.36±1.06	0.526
TISS total			11593.22±11693.03	38364.64±42240.21	0.383
APACHE II			9.62±3.29	9.14.±3.40	0.455
CRP			3.41±9.02	2.33±14.58	0.130
CRP 1 day after OP			35.66±19.97	38.89±24.24	0.541
Airways	pts	1 (0.36%)	1 (0.40%)	0 (0.00%)	0.767
Mechanical ventilation	pts	1 (0.36%)	1 (0.40%)	0 (0.00%)	0.767
Artery catheters	pts	32 (11.68%)	29 (11.51%)	3 (13.64%)	0.766
Central venous catheter	pts	2 (0.73%)	2 (0.73%)	0 (0.00%)	0.675
Urine catheter	pts	263 (95.99%)	243 (96.43%)	20 (90.91%)	0.423
Hospital wound complications	pts	27 (9.85%)	15 (5.95%)	12 (54.55%)	**<0.001**

*ASA* American Society of Anesthesiologists, *NICU* neurointensive care unit, *TISS* Therapeutic Intervention Scoring System, *APACHE* Acute Physiology and Chronic Health Evaluation, *CRP* C-reactive protein, *OP* operation

However, in the SSI group, we found more wound complications of other etiologies (such as dehiscence, secretion, seroma, or haematoma) (Table 4). These complications together with incidence during the warm season (June, July, and August) were found to be the only significant predictors of SSI according to our multivariate logistic regression analysis (Table 5).

**Table 4:** Characteristics of surgical site infection

Number	SSI	Occurrence interval	Wound complication	Microbiology
1	Superficial	30 days	Dehiscence	0
2	Superficial	30 days	Dehiscence	0
3	Superficial	30 days	Dehiscence	0
4	Superficial	30 days	Secretion	0
5	Superficial	30 days	Dehiscence	*Staphylococcus aureus Methicillin-sensitive*
6	Superficial	30 days	Dehiscence	0
7	Superficial	30 days	Dehiscence	*Staphylococcus aureus Methicillin-sensitive*
8	Superficial	30 days	Dehiscence	*Staphylococcus aureus Methicillin-sensitive*, *Streptococcus viridans*
9	Superficial	30 days	Dehiscence	0
10	Superficial	30 days	Dehiscence	*Staphylococcus aureus Methicillin-sensitive*, *Peptococcus Peptostreptococcus*,
11	Superficial	30 days	Secretion	0
12	Superficial	30 days	Secretion	Negative
13	Superficial	30 days	Dehiscence	*Staphylococcus aureus Methicillin-sensitive*, *Klebsiella pneumoniae*
14	Superficial	30 days	Seroma	0
15	Superficial	30 days	Dehiscence	*Staphylococcus aureus Methicillin-sensitive*
16	Superficial	30 days	Dehiscence	*Streptococcus alfa*, *Propionibacterium*
17	Deep	30 days	Dehiscence	*Staphylococcus Coagulase-negative*
18	Deep	30 days	Dehiscence	*Enterococcus faecalis*, *Pseudomonas aeruginosa*, *Peptostreptococcus*
19	Deep	30 days	Secretion	*Staphylococcus aureus Methicillin-sensitive*, *Acinetobacter baumanii*
20	Deep	1 year	Secretion	*Staphylococcus aureus Methicillin-sensitive*
21	Deep	30 days	Dehiscence	0
22	Organ	30 days	Haematoma	*Staphylococcus coagulase-negative*

**Table 5:** Multivariate logistic regression analysis of surgical site infections (*CL* confidence limit)

Multivariate analysis				
Surgical site infections	Odds ratio	Lower CL 95%	Upper CL 95%	***p*** value
Hospital wound complication	20.40	7.32	56.85	<0.001
Warm season	2.92	1.03	8.27	0.044

## Discussion

The incidence of SSI is an important mark of quality management in every surgical procedure. Since these infections are preventable, it is important to take an interest in their monitoring [2]. SSIs can worsen the final results of operations, and additionally in spinal surgery, a patient’s mobility can be affected due to the close proximity of the spinal cord and neural structures. This will raise the costs of care for the spine operation.

Since new therapeutic approaches are limited, the basis of SSI management is prevention. This means maintaining an aseptic environment, a standard which is followed closely in the operating theatre. This standard is also important throughout every stage of the postoperative period, and especially in the initial phase until the wound has healed.

The biggest challenge to the implementation of a preventative care protocol is compliance of the entire team of doctors, nurses, and technicians.

An important component of our SSI prevention strategy was the correct antibiotic prophylaxis [1]. Antibiotic prophylaxis is based on the principle of eliminating any bacterial contaminant by administering a suitable antibiotic so that it is present in the surgical site, even in blood clots, in an effective bactericidal concentration throughout the entire operation. One common mistake which has significant epidemiological consequences is the inappropriate prolongation of antibiotic prophylaxis. All our prophylaxes were short-term, with the exception of 12 (4.20%) of our 286 consecutive patients, who received prolonged antibiotic prophylaxis. These 12 patients were excluded from our study for this reason. Another common error that substantially impairs the effectiveness of antibiotic prophylaxis is the incorrect timing of preoperative administration. We resolved this issue by giving antibiotics immediately in the preoperative prep-room, thus achieving the appropriate level of protection at the time of incision. Last but not least, failure to administer appropriate doses when operations are prolonged can result in excessive or insufficient prophylaxis.

To interpret the quality and effectiveness of antibiotic prophylaxis properly, patient populations should be stratified according to risk and outcomes, and interpretation should take into account the influence of other risk factors. For each procedure, process indicators and audit methodology should be defined in the quality assessment of antibiotic prophylaxis. In order to draw a statistically meaningful conclusion, a minimum of 100 homogenous procedures should be evaluated. Our study population of 274 patients (defined by 6-year period) fulfils this criterion with a high safety margin. For each operation, the patient’s weight, antibiotic administered, route of administration, dose rate, exact time of dosing, time and extent of any additional doses during the operation, time of the end of the operation, overall length of the operation, and the number of doses of antibiotic administered at the end of the procedure were recorded.

Incidence of SSI in spine surgery varies from 2 to 13% [3–10]. This wide variation is because a large proportion of the studies are of a retrospective design, the criteria for the definition of SSI is inconsistent, and a lack of meticulous reporting, as cited in *Spine* (Boody and Vaccaro, 43) [9]. Our results are based on prospectively collected data, a consecutive population, and the internationally accepted definition of SSI according to Horan et al. [11]. During this 6-year monitoring period, we identified 22 patients with SSI, which is at the upper limit (8.03%) of the results reported in literature. However, we emphasize that we included every single incidence of SSI, including borderline cases; we decided to include these due to our experience of non-purulent secretion with positive microbiology. This result reflects genuine evaluation and accurate reporting. The vast majority of complications were classified as superficial and there were very few deep (1.82%) and organ complications (0.36%). Since all infections in the wound (22 patients) were preceded by a non-infective wound complication, it is evident that these complications, which had various causes including the quality of wound care, are a significant risk factor. Established procedural methods in the preoperative and perioperative period confirm a high degree of preventability of SSI [2]. For this reason, it is important to carefully monitor all such non-infectious wound complications.

Another significant factor (in fact a leading factor in cases of delayed SSI) appears to be patient compliance. However, this factor is extremely hard to describe using numerical methods and it was not evaluated in our study.

Contrary to the prevailing conclusions in literature [12–15], some of the anticipated risk factors were not confirmed by our study, namely diabetes mellitus, the use of corticosteroids, transfusions, or ulcer prophylaxis. Concerning diabetes mellitus, we attribute the result to our strict maintenance of normoglycemia in our patients. Two reasons for the insignificant influence of corticosteroids as a risk factor may be the short duration of immunosuppressive therapy during the preoperative and postoperative period (mean duration was 2.14±1.17 days) and the size of the dose administered. The prevailing corticosteroid was hydrocortisone in a substitute dose (150–300 mg), which was not indicated as a risk factor for SSI in the results. The insignificant influence of transfusions can probably be attributed to our transfusion strategy with a low trigger of haemoglobin levels (70 g/L), which resulted in fewer transfusion cases. This study found that besides non-infectious wound complications as a general category, the only significant risk factor was operation during the warm season, in our region from June to August. In these months, the local average temperature is 20.9°C in June, and 18.7 in both July and August. The reason for an increase in SSI during the warm season is primarily due to the lack of air-conditioning in our wards at the time of the study. The higher ambient temperature during the summer leads to increased sweating of the skin and results in less favourable conditions for wound healing, requiring more frequent re-dressing of the wound. Skin irritation is more likely, leading to decreased compliance by some patients (keeping the dressings clean and avoiding mechanical stimuli to the wound). However, this explanation is purely observational, as such factors are nearly impossible to evaluate numerically.

There is a slight increase in the rate of SSI during January and February. We did not identify any objective factors for such an increase. The only possible explanation we could think of is speculative in its nature. Considering our system of reimbursement, the surgeons always face restrictions of budget and resources at the end of the fiscal year. They admitted they tend to operate on patients with more favourable radiological findings and less risk in general terms during December. They also tend to postpone more difficult cases to the beginning of the new fiscal year. Such selection is based on personal, nonparametric experience and subjective evaluation. Our assumption is further supported by zero SSI during the final month of the year; however, we have no means for any kind of numerical evaluation.

Our study has several limitations. We do not evaluate some factors of potential significance, namely smoking and nutrition. Despite the majority of elective operations, these factors cannot be assessed properly due to lack of data on admission and the short period before surgery. All the patients had glucose values tested during the period before the operation; however, immediate preoperative values were measured only in patients with diabetes mellitus.

## Conclusions

Contrary to the prevailing literature, our study on a population of planned thoracic or lumbar spine surgery patients with short antibiotic prophylaxis hospitalized postoperatively in the NICU did not identify corticosteroids, diabetes mellitus, or transfusions as risk factors for SSI. Our study concludes that any kind of non-infectious wound complication and operation during the warm season represent independent risk factors for developing such infections.

## Data Availability

The datasets obtained during this study are available from the corresponding author on reasonable request.

## Abbreviations

APACHE: Acute Physiology and Chronic Health Evaluation
ASA: American Society of Anesthesiologists
BMI: Body mass index
CL: Confidence limit
CRP: C-reactive protein
NICU: Neurointensive care unit
OP: Operation
SSI: Surgical site infection
TISS: Therapeutic Intervention Scoring System
